# Immunotherapies in Non-Hodgkin’s Lymphoma

**DOI:** 10.3390/cancers13143625

**Published:** 2021-07-20

**Authors:** Christine Bezombes, Patricia Pérez-Galán

**Affiliations:** 1 Centre de Recherches en Cancérologie de Toulouse, INSERM UMR1037, CEDEX 1, 31037 Toulouse, France; 2 Université Toulouse III Paul-Sabatier, CEDEX 9, 31062 Toulouse, France; 3 ERL 5294 CNRS, CEDEX 4, 31055 Toulouse, France; 4 Institut Universitaire du Cancer-Oncopole de Toulouse, CEDEX 9, 31059 Toulouse, France; 5 Laboratoire d’Excellence ‘TOUCAN-2′, CEDEX 1, 31037 Toulouse, France; 6 Institut Carnot Lymphome CALYM, 69495 Pierre-Bénite, France; 7 Department of Hemato-Oncology, Institut d’Investigacions Biomèdiques August Pi i Sunyer (IDIBAPS), 08036 Barcelona, Spain; 8 Centro de Investigación Biomédica en Red-Oncología (CIBERONC), 28029 Madrid, Spain

Immune-based therapies mobilize the immune system to promote or restore an effective antitumor immune response. The types of immunotherapies available for the non Hodgkin’s lymphoma range from antibody-based (monoclonal or bispecific antibodies, immune checkpoint inhibitors), to cell-based therapies (CAR-T cells). This special issue proposes an overview of the last immunotherapies developed to target and eradicate B cell lymphomas and highlight the role of immune system in non-Hodgkin’s lymphoma pathogenesis and therapy.

Non-Hodgkin’s lymphoma (NHL) holds the 10th position in the rank of the most frequent cancers in terms of new cases and total of deaths in 2020 (World Health Organization (WHO)). More than 80 specific NHL subtypes have been identified and are categorized by the WHO according to the cell origin (B, T, and NK cells), their morphology, immunophenotype, genetic features, and clinical features. Aggressive and indolent lymphoma can be separated according to the evolution of the disease. The most representative cases are diffuse large B cell lymphoma (DLCBL, 27%), chronic lymphocytic leukemia (CLL, 23%), and follicular lymphoma (FL, 14%) [1].

The types of immunotherapies available for the NHL range from antibody-based to cell-based therapies. There are currently ten FDA-approved immunotherapy options for lymphoma.

Initially treated with radiation and diverse combinations of chemotherapeutic agents, the introduction of hematopoietic stem cell transplantation (HSCT) constituted the first form of immunotherapy that was first applied to treat acute leukemia in 1957 [2] and then applied to NHL in the 1980s after high-dose radio-chemotherapy.

The following breakthrough was the development of rituximab, an anti- CD20 monoclonal antibody approved by FDA in 1997, which, in combination with chemotherapy revolutionized the treatment and the prognosis of almost all CD20+ B -cell malignancies. This discovery established the proof-of-principle that the immune system can be exploited to eliminate cancer cells [3,4,5]. However, disease resistance or relapse after successful initial therapy and declining efficacy of subsequent rounds occurs. Thus, a number of second and third generation anti-CD20 antibodies have been developed, such as ofatumumab, ublituximab, and obinutuzumab with specific mechanisms of action [6]. So far, obinutuzumab-based immunochemotherapy has demonstrated superiority over rituximab-based immunochemotherapy for first-line treatment (GALLIUM study) [7].

Moreover, a number of therapeutic monoclonal antibodies against alternative surface targets have been developed to treat CD20- lymphomas, such as anti-CD19, anti-CD79b or anti-CD22 in some cases coupled to toxins/chemotherapeutics.

However, a high incidence of disease relapses occurs after these treatments, and innovative and powerful new immunotherapies have been developed. While conventional mAbs can eliminate target cells *via* several mechanisms including antibody-dependent cell cytotoxicity (ADCC), antibody-dependent cell phagocytosis (ADCP) or complement-dependent cytotoxicity (CDC), they do not exploit the powerful cytotoxic machinery of T -cells that eradicates tumor cells by the formation of an immune synapse and the release of granzyme B/perforin vesicles. These types of antibodies represent a fast- growing area of immunotherapy, with over 100 different bispecific antibody (bsAbs) formats [8,9,10]. The majority of bsAbs in advanced stage clinical development for lymphoma are those containing the Fc region, that prolongs its half-life and it is usually re-engineered to reduce excessive cytokine release from recruited T cells and to minimize off-target toxicity such as T-cell mediated hepatotoxicity.

Blinatomumab (CD3 × CD19) belongs to the first generation of bsAbs, while approved for acute lymphocytic leukemia, it is currently being evaluated in several clinical trials for R/R DLBCL with overall response rate (ORR) ranging from 37–55%. Second generation bs-mAbs include mosunetuzumab, glofitamab, and epcoritamab. These antibodies are under investigation both in the R/R setting, including challenging scenarios such as chimeric antigen receptor T cells (CAR-T)-therapy relapsed, and in the front-line in combination with standard chemo-immunotherapy or replacing anti-CD20 antibodies.

One key difference among bsAbs is the number of Fab arms within each antibody. Mosunetuzumab (CD3 × CD20) has a 1:1 CD3:CD20 ratio of Fab arms, whereas glofitamab (CD3 × CD20) has a 1:2 CD3:CD20 ratio, facilitating higher avidity of the binding of the T-cell *via* two CD20 antigens on the target B-cell simultaneously. Notably, the CD20 Fab of glofitamab is derived from the second generation CD20 monoclonal antibody, obinutuzumab, which itself was glycoengineered to enhance binding and increase ADCC. These differential features may lay at the basis of the variety of results observed in clinical trials [11].

Mosunetuzumab, is being tested in both the R/R NHL setting as well as first line, either as a single agent or in combination with CHOP-like regimens and polatuzumab vedotin. In patients with aggressive NHL, the ORR was 37.4%, while in indolent NHL the ORR reached 62.7%. Very good responses (75.9%) were observed in FL with progression within 24 months of first treatment (POD24). Mosunetuzumab is also under investigation for frontline therapy, either as a monotherapy or in combination with chemotherapy.

In the case of glofitamab, it is administered after obinutuzumab in order to occupy CD20 on the surface of lymphoma cells as well as depleting peripheral B-cells to reduce the risk of cytokine release syndrome (CRS). Results for initial phase one trials are encouraging with ORR superior to 50% in aggressive NHL and higher than 80% in indolent NHL. A number of trials are ongoing both in the relapsed setting in combination with several agents (i.e., anti-CD79b, anti-PDL-1, or chemotherapy) and in front-line in combination with CHOP, R-CHOP or obinutuzumab-CHOP. Finally, epcoritamab (DuoBody- CD3 × CD20) induces potent T-cell-mediated killing in NHL (FL, MCL and DLCBL) pre-clinical models irrespective of prior treatments and provides opportunities for subcutaneous dosing [12]. Preliminary results in a phase one trial indicates that this subcutaneously-administered Ab is able to engage response in 100% of DLBCL, MCL and FL cases at the highest dose tested. Altogether, these studies are very encouraging and open new perspectives as its combination with mAbs, as shown for glofitamab and obinutuzumab, has shown very promising results in R/R NHL [13].

In the last years, we have also attested an explosion in the development of two additional types of immunotherapies: the adoptive cell therapy of CAR-T cells and the immune checkpoint (ICP) inhibitors. CAR-T cells have emerged as a new pillar in the treatment of NHL. The generation of CAR-T cells involves the collection of autologous peripheral blood mononuclear cells, isolation of T cells, transduction of a transgene encoding for the CAR, expansion to reach a critical number of T cells, and formulation of the clinical-grade therapeutic products [14]. First-generation CARs included a tumor antigen binder, complexed *via* a linker and a transmembrane (TM) domain to the ζ-chain of the CD3. Second-generation constructs result from adding a co-stimulatory motif such as CD28 or 4-1BB to a first-generation CAR scaffold. CARs using CD28 promotes activation-induced cell death (AICD) [15] and appear to be associated with more rapid in vivo expansion but lower long-term persistence due to differentiation to effector memory phenotype and possibly more rapid metabolic exhaustion compared with CARs using 41-BB [16,17,18,19]. Third-generation CARs include more than one co-stimulatory domain.

The first CAR-T cell-based therapies were developed to target the CD19 antigen, and several of the second generation CARTs including CD28 or 4-1BB as costimulatory domain have progressed in the clinical development.

In this regard, based on ZUMA-1 and JULIET studies, two autologous anti-CD19 CAR T-cell therapies were approved by 2018 for R/R aggressive B-NHL in the third line and beyond: axicabtagene ciloleucel (axi-cel) and tisagenlecleucel (tisa-cel) [20,21]. More recently in 2021, the TRANSCEND NHL-001 study resulted in the approval of lisocabtagene maraleucel (liso-cel) in R/R NHL [22]. Regarding other incurable lymphomas, ZUMA-2 [23] allowed the approval of brexucabtagene autoleucel (brexu-cel) [24] for the treatment of R/R MCL in 2020 and the ZUMA-5 study for axicel resulted in its approval in R/R FL in 2021.

Currently, 126 clinical trials of CAR-T in NHL (including DLBCL, MCL, and FL) are ongoing mostly in United States and East Asia. Emerging real-world evidence seems to confirm the promising results. However, there is considerable toxicity limiting their general applicability pushing forward a development of simultaneously more effective and safer CARs. Possibilities to improve the outcomes include the combination with immunotherapy such as ICP inhibitors, kinase inhibitors or immunomodulators [25]. The identification of resistance factors will also help to improve the efficacy of these new cell-based immunotherapies. For instance, lack of persistence of the CAR T cells, loss of the CD19 antigen, and upregulation of ICP such as PD-L1 on the tumor cells have be reported [26]. The composition of the tumor microenvironment (TME) prior to therapy is emerging as a predictor for the success of treatment [27]. In addition, clinical factors, such as high tumor burden, high lactate-dehydrogenase and poor performance have also been shown to correlate with lack of response.

The third generation (3G) of CAR-T cells incorporate two co-stimulatory signals such as CD28/4-1BB thus increasing phosphorylation of signaling proteins which result in better expansion in in vitro and in preclinical models [28]. Applied to patient with B cell malignancies, 3G CARs promote a faster expansion and a longer persistence than second generation CARs, especially when the tumor burden is low [29]. Prospective clinical trials are needed to confirm these findings. Other 3G CARs including CD28/OX40 or ICOS/4- 1BB are also tested with inferior or comparable efficacy than their second generation counterpart [30,31].

Moreover, novel approaches and the next generations (fourth and fifth) of CARs are under development. These include the transduction of cells with two different CARs, both equipped with co-stimulation domains or one CAR harboring an inhibitory domain. Moreover, in order to combat the immunosuppressive TME, CAR-T cells redirected for universal cytokine killing (TRUCKs) [32] are equipped with an inducible cytokine expression cassette, such as IL-12 or IL-15 [33,34]. Upon antigen engagement, these armored CAR-T cells secrete IL-12 in a locally restricted manner, and recruit both primary adaptive and innate immune cells, such as cytotoxic T cells and NK cells, to the tumor sites. In summary, CAR-T therapy has an established position in the treatment paradigm of lymphoma with a long road ahead. However, how best to sequence and combine these products with current treatment and how to overcome issues of resistance and toxicity remain pivotal questions in the field.

Finally, we would like to introduce the ICP inhibitors that serve to maintain self- tolerance and prevent autoimmunity and that further expanded the list of tumor immune-escape mechanisms [10,35,36]. These molecules are present on the cell surface of T cells and antigen-presenting cells controlling the efficacy of the formed immune synapse and therefore modulate the intensity and the duration of TCR-dependent T-cell responses [37]. Immune evasion mechanisms are present both in tumor cell and in the TME interfere limiting tumor recognition and sufficient effector cell infiltration thus supporting tumor cell survival [38].

It is noteworthy that, similarly to solid cancers, NHL is also categorized in inflamed and non-inflamed tumors [39]. An inflamed TME lymphoma is enriched in infiltrating immune cells, including CD4+ and CD8+ T cells, and shows activation of oncogenic signaling pathways (i.e., NF-κB and NOTCH) as a consequence of recurrent mutations. Conversely, a non-inflamed TME lacks T cells infiltration due to oncogenic alterations (i.e., CREBBP, EZH2, or B2M) that may contribute to immune escape and inhibition of effective immune cells recruitment. This immune landscape of lymphoma is a critical point to predict the response to immunotherapy and to design new therapeutic approaches [39,40,41].

While Hodgkin’s lymphoma (HL) has followed the story of success in solid tumors regarding anti-PD1, this has been not the case in NHL. Two anti-PD1 mAbs nivulomab or pembrolizumab obtained FDA approval in 2016 for R/R HL. The underlying mechanism for this success is the recurrent genetic alterations of the *PD-L1*/*PD-L2* locus on chromosome 9p24 in HL, resulting in high PD-1 ligand (PD-L1) expression on Hodgkin Reed-Sternberg cells, together with PD-L1 expression in the HL microenvironment [42]. However, the benefit observed in HL has not been translated to most of the NHL subtypes, where the expression of PD-1 ligands (PD-L1 and PD-L2) is not governed by genetic aberrations, and in most of the cases, it is mainly restricted to the TME and most commonly macrophages. In NHL, the interplay between the tumor cell and the immune infiltrates may be governed by ICP that differ among NHL subtype and evolves during disease progression. In the last years, great efforts are being devoted to characterize specific immune scenarios in each lymphoma subtype, using high content technologies such as mass cytometry and single cell RNA sequencing [43,44]. The number of ICP is quite large and the best characterized so far in NHL are PD-1/PD-L1, PD-L2, LAG-3, TIM3, CTLA-4, and TIGIT.

TIGIT is highly expressed in CD4 and CD8 T cell from NHL patients, commonly in co-expression with exhaustion markers such as PD-1 and CD244. While TIM-3 and LAG-3 seem to be expressed at moderate levels in most NHL [45], they also go together with PD-1 and play a role in immunosuppression in NHL. CD8+TIM-3+ cells show defective cytokine production upon TCR engagement, despite the presence of *ex vivo* markers of lytic granules, as described for FL [46], and co-targeting of PD-1 and LAG3 enhances the function of intratumoral CD8+ T cells. Likewise, LAG3, TIM-3 and TIGIT have been related with inferior outcomes in FL [47].

This knowledge has provided the basis to design successful ICP combinations in NHL using the already well-developed armamentarium. Since 2012, a frenetic race involving anti-ICP mAbs in NHL treatment was engaged with a continued increase in the number of clinical trials to peak in 2018. These trials include the combination of antibodies targeting several ICP (i.e., PD-1, CTLA-4, CD47 and 4-1BB) with therapeutic antibodies (i.e., anti-CD20), kinase inhibitors (PI3Ki and BTKi) or HDAC inhibitors [35]. Moreover, clinical trials associating CAR-T cells and PD-1/PD-L1 blockade are also on going (NCT029226833 and NCT02706405).

A complementary approach is to target the immune checkpoint activators. This includes anti-41BB, anti-CD40, and anti-CD80 antibodies that are currently under investigation in combination mainly with anti-CD20.

This special issue will cover both basic and (pre)-clinical research on immunotherapies on B cell lymphoma by increasing our understanding of the role of immune system in B-cell lymphoma development and thus opening up novel therapeutic perspectives for NHL patients.
